# Intercostal lung herniation secondary to thoracotomy: a case report

**DOI:** 10.11604/pamj.2020.36.39.20054

**Published:** 2020-05-27

**Authors:** Samira Mhamdi, Ines Aouini, Salsabil Daboussi, Houaida Mahfoudhi, Mehdi Ben Lassoued, Manel Kallel, Zied Moetamri, Chiraz Aichaouia, Islem Mejri, Mohsen Khadhraoui, Rzaieg Cheikh

**Affiliations:** 1 Pulmonology Department, Military Hospital, Tunis, Tunisia; 2 Cardiology Department, Military Hospital, Tunis, Tunisia; 3 Emergency Department, Military Hospital, Tunis, Tunisia

**Keywords:** Hernia, lung, chest, surgery

## Abstract

Intercostal lung herniation is defined as a protrusion of the lung parenchyma through a defect in the intercostal muscles between adjacent ribs. The authors report a case of intercostal pulmonary hernia in a 45-year-old male patient, with smoking habit (30 packs-year), presented to the emergency department with dyspnea. He had the history of pulmonary emphysema complicated with a total right pneumothorax in 2015 treated by mini-thoracotomy with bullectomy and pleural abrasion. In 2019, he was admitted to hospital for left chest pain. The computed tomography (CT) scan of the chest revealed a bilateral emphysema with intercostal lung hernia through the fourth intercostal space the patient underwent, a left thoracotomy with repair of the intercostal muscle defect. He was discharged from hospital free of complications.

## Introduction

Intercostal lung herniation is defined as a protrusion of the lung parenchyma through a defect in the intercostal muscles between adjacent ribs. It presents as a soft, subcutaneous nontender bulge visible on coughing or straining [1]. Acquired lung hernias are usually post trauma or post-surgery complications. They can arise immediately after the trauma or surgery, or be discovered long after the initial injury [2]. They may be asymptomatic, or may be revealed by pain and hemoptysis if incarceration or strangulation is present. We present a case report of post thoracotomy intercostal lung herniation.

## Patient and observation

**Figure 1:**
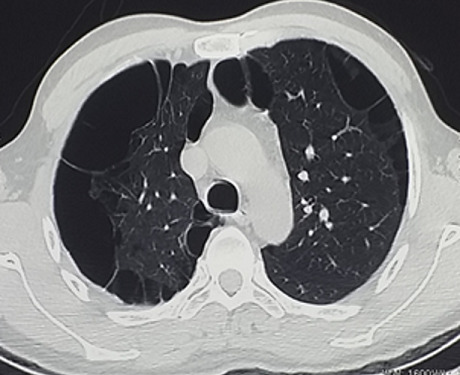
Computed tomography scan: pulmonary emphysema

**Figure 2:**
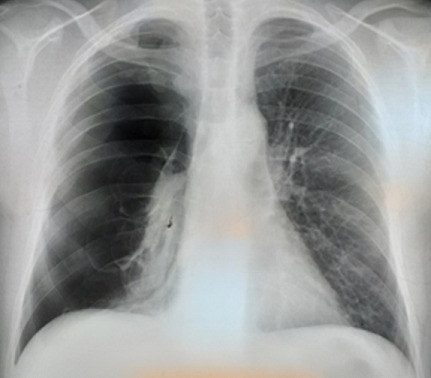
Total right pneumothorax

A 45-year-old male patient, with smoking habit (30 packs-year), presented to the emergency department with dyspnea on exertion, cough and left chest pain for five days. He had a history of pulmonary emphysema and his CT scan revealed a bilateral paraseptal and centolobular emphysema (Figure 1). It was complicated with a total right pneumothorax in 2015 (Figure 2) treated by mini-thoracotomy with bullectomy and pleural abrasion. In 2016, he had a total left pneumothorax treated by thoracotomy with bullectomy, his postoperative course was simple and his pulmonary function tests, were normal, demonstrated a FEV1 of 2.35 liters (71% predicted) and FCV of 2.69 liters (68% predicted).

**Figure 3:**
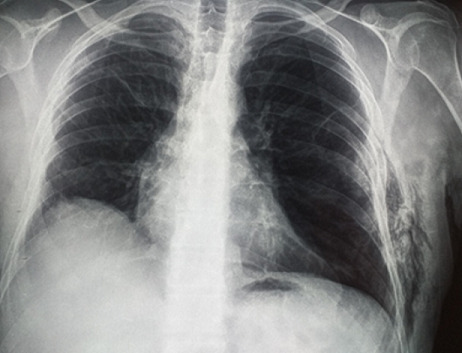
Chest X-ray: parietal air image

**Figure 4:**
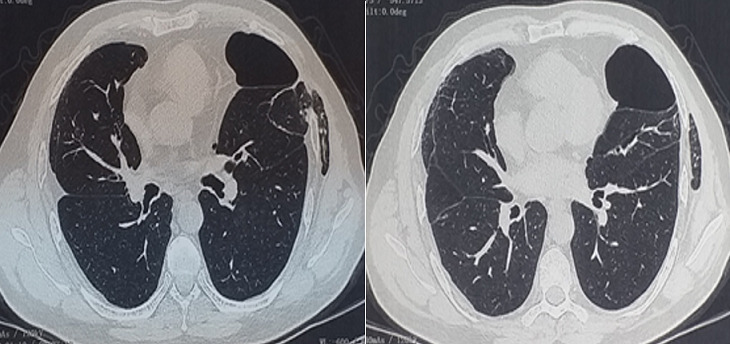
Computed tomography scan: intercostal lung hernia

In 2019, he was admitted to the hospital for left chest pain. Physical examination revealed subcutaneous crepitation and emphysema at the level of the 4^th^ and 5^th^ ribs. Laboratory values were normal. Chest X-ray showed a parietal subcutaneous gas along the left lower chest wall (Figure 3). The CT scan of the chest revealed a bilateral emphysema with an intercostal lung hernia through the fourth intercostal space (Figure 4). The patient underwent a left thoracotomy with repair of the intercostal muscle defect. His postoperative course was uneventful and he was discharged from the hospital free of complications.

## Discussion

Thoracic hernias are rare conditions characterized by the protrusion of lung parenchyma outside the thoracic cage [3]. Approximately 66% of thoracic hernias push through a weak area in the chest wall, usually acquired after severe thoracic trauma; the rest of hernias involve the neck and, very rarely, the diaphragm [4, 5]. The first reported case of thoracic herniation was described by Roland in the 15^th^ century. Thoracic hernias were classified by Morel-Lavallée depending on their localization and etiology [4]. Several cases have been reported in the literature [4, 6]. Minai classified the cases by etiology, reporting 64 cases of spontaneous thoracic hernias [6]. Ross and Burnett postulated a lower incidence of this etiology by reviewing the existing literature that revealed trauma as the underlying cause in most of cases [7].

To-date, the most common cause of acquired thoracic herniation is traumatic, with associated injuries such as rupture of great vessels, pneumothorax or hemothorax [8]. In patients with spontaneous acquired thoracic herniation, one of the frequent causes is chronic obstructive pulmonary disease. Post surgery hernias are also frequent. Connective tissue disorders and congenital abnormalities in the chest wall may cause congenital lung herniation [9]. Frequently-associated symptoms with this condition include pain, persistent cough, shortness of breath and hemoptysis, nevertheless, many of these hernias can be completely asymptomatic [10]. Imaging studies play an important role in the diagnosis of thoracic herniation. A chest X ray helps in making the diagnosis. CT scan with intravenous contrast is considered the gold standard for thoracic herniation, since it evaluates the defect protruding out of the thoracic wall and the viability of the lung parenchyma [11].

There is significant controversy as to the ideal management of these patients. Each case must be individualized depending on the characteristics of the patient and of the affected area [12]. Most of the thoracic hernias resolve with a conservative management but, hernias with incarceration and strangulation are candidates for reduction and repair of the defect and removal of non-viable tissue [13, 14].

## Conclusion

Lung herniation should be considered in the differential diagnosis of patients who present with localized pain and subcutaneous emphysema after thoracic surgery. CT imaging and surgical consultation should be evaluated at an early stage. Conservative management is usually enough for mild and moderate herniation but for larger herniations a surgical approach may be necessary.

## Competing interests

The authors declare no competing interests.
